# Isolation, characterization, and molecular identification of *Candida* species from urinary tract infections

**DOI:** 10.18502/cmm.5.2.1159

**Published:** 2019-06

**Authors:** Amin Gharanfoli, Elaheh Mahmoudi, Roya Torabizadeh, Farzad Katiraee, Saeid Faraji

**Affiliations:** 1 Student Research Committee, School of Medicine, Alborz University of Medical Sciences, Karaj, Iran; 2 Department of Mycology, School of Medicine, Alborz University of Medical Sciences, Karaj, Iran; 3 Department of Bacteriology, School of Medicine, Alborz University of Medical Sciences, Karaj, Iran; 4 Department of Pathobiology, School of Veterinary Medicine, University of Tabriz, Tabriz, Iran; 5 Statistical Researcher, Faculty of Electronic Unit, Islamic Azad University, Tehran, Iran

**Keywords:** *Candida* species, Predisposing factors, Urinary tract infections

## Abstract

**Background and Purpose:**

*Candida* species are reportedly the most common human fungal pathogens. The incidence of urinary tract infections (UTIs) caused by *Candida* pathogens has increased in recent decades. However, such infections rarely occur in the absence of any predisposing factors. Regarding this, the aim of the present study was to identify the *Candida* species causing UTIs and determine the predisposing factors for candiduria.

**Materials and Methods:**

The current study was conducted on 1,450 urine samples obtained from patients suspected of UTI. Out of this number, 19 cases were candidiasis, and 2 cases were mixed infections caused by bacteria and fungi. *Candida* species were diagnosed differentially using the germ tube test, colony staining on CHROMagar medium, intracellular beta-glucosidase enzyme activity, and glucose absorption pattern. Then, the colonies with the same morphology were confirmed by the DNA sequencing of internal transcribed spacer regions.

**Results:**

According to the results, 38%, 28.6%, 14.3%, and 9.5% of the isolates were identified as *C. albicans, C. glabrata, C. tropicalis, *and* C. kefir*/*C. krusei,* respectively. The presence of one or more predisposing factors was proved in all patients in whom diabetes was the most prevalent predisposing factor (21.1%).

**Conclusion:**

Based on the obtained results, *C. albicans* species was the most prevalent fungal species. In addition, urinary fungal infections were less prevalent than bacterial urinary infections.

## Introduction

Urinary tract infection (UTI) is one of the most commonly diagnosed infections in both nosocomial and community-acquired infections [1]. Bacteria and fungi are the etiologic agents of UTI [2]. There is some evidence indicating a decrease in the percentage of *E. coli, Proteus *species*, *and* Pseudomonas* species and an increase in the percentage of UTI caused by fungi, Streptococcus agalactiae, and *Klebsiella pneumoniae* [2-4]. The incidence of UTIs caused by fungal species, especially *Candida* species (candiduria), has increased by 2-3 times in recent decades [4,5]. 

Candiduria is classified into asymptomatic and symptomatic forms. Most of the patients who excrete *Candida* in their urine are asymptomatic. On the other hand, symptomatic candiduria is seen in patients with renal candidiasis, pyelonephritis, cystitis, epididy-morchitis, and prostatitis [6]. Candiduria may result from deep fungal infections. Studies show that in most cases with a reported growth of *Candida* in the urine cultures, the conditions are transient and have no association with systemic infection [6-7]. However, 10% of blood infections caused by *Candida* result in candiduria [8].* Candida* yeast can cause urinary tract and renal infections the common symptoms of which include pain, dysuria, micturating, hematuria, and pyuria [7].

These infections rarely occur in the absence of any predisposing factors. In this regard, diabetes, long hospital stay, organ transplantation, recurrent bacterial infections, antibiotic use, aging, and use of catheter are among the important predisposing factors for these infections [9, 10]. The most common risk factor for candiduria is the use of a catheter, especially in the patients admitted to intensive care units [9]. In this respect, in a study investigating UTI, 26.5% of people who were using catheter developed UTI due to *Candida* species [10]. Catheters provide a surface for the adhesion and colonization of organisms into the bladder, thereby causing mucosal irritation [11].

Among *Candida* species, *C. albicans* is the main pathogen isolated from most of the clinical samples [12]. However, in an international surveillance study, *C.* *glabrata* was introduced as the predominant species [13]. Other *Candida* species isolated from UTIs include *C. tropicalis *and* C. parapsilosis, C.* *krusei*, *C. guilliermondii*, *C. kefir,* and *C.* *parapsilosis *[14, 15]. A few studies with small sample size have addressed the epidemiology and risk factors of candiduria, as well as species distribution in this disease. 

It seems that the definition of candiduria is problematic due to the inability to distinguish the colonization of infections [6]. The repeat urine cultures and identification of the foci of infection by imaging studies are effective approaches for the establishment of differential diagnosis in patients with candiduria [16].

Despite the low rate of mortality in candiduria cases, it is crucially important to identify *Candida *strains at the species level because of their difference in antifungal susceptibility patterns [17, 18]. For example, the use of fluconazole therapy can lead to the development of UTI due to *C. glabrata *[19]. The identification of the microbial agent of the infection is important for the proper treatment and prevention of the disease from becoming chronic [18]. On the other hand, some studies have suggested that the management of UTIs by the eradication of predisposing factors are more effective than their treatment [19, 20]. Regarding this, the aim of the present study was to detect the prevalence rate of candiduria and determine the role of predisposing factors in causing this infection. 

## Materials and Methods

***Collection and maintenance of isolates***

During a period of 4 months, 1,450 urine samples were collected from hospitalized patients and outpatients, who were suspected of UTI, in Shahid Heidari Hospital, Tehran, Iran. The urine samples were transferred to a medical mycology laboratory in sterile containers. 

***Differential tests for Candida species***

- ***Examination of the color and form of colonies on CHROMagar Candida medium***

For the initial identification of *Candida* species, 10 μL of each urine sample was inoculated on chromogenic *Candida* agar (bioMerieux, France) [5]. In addition, *Candida* species were diagnosed differentially based on the germ tube test [12], intracellular beta-glucosidase enzyme activity, and glucose absorption pattern [20].

- ***Amplification of internal transcribed spacer regions***

The internal transcribed spacer (ITS) of ribosomal DNA was amplified using two primers, namely ITS4 and ITS5. Genomic DNA was isolated by phenol-chloroform and isoamyl-alcohol method according to the reference protocol [21]. Polymerase chain reaction (PCR) was performed by general fungal ITS4 and ITS5 primers that amplify the regions with 650 bp fragment length, encoding ITS1 and ITS2 (ITS4:5΄-TCCTCCGCTTATTGATATGC-3΄, ITS5: 5΄-GGAAGTAAAAGTCGTAACAAGG -3΄).

- ***DNA sequencing of internal transcribed spacer regions***

All PCR-amplified products were sequenced by the Applied Biosystems 3730 XL Bioneer (Korea) using ITS4 primer. Sequence search was performed through local blast with a molecular database maintained at the NCBI (Library of Medicine, Bethesda, MD, USA; http://www.ncbi.nlm.nih.gov/BLAST/). 

***Statistical analysis***

The rate of candiduria was calculated in SPSS software, version 15 (SPSS Inc., Chicago, IL, USA). The Chi-square test was used to prove the probable association between predisposing factors and candiduria. A *p-value* less than 0.05 was considered statistically significant. 

## Results and discussion

**Figure 1:**
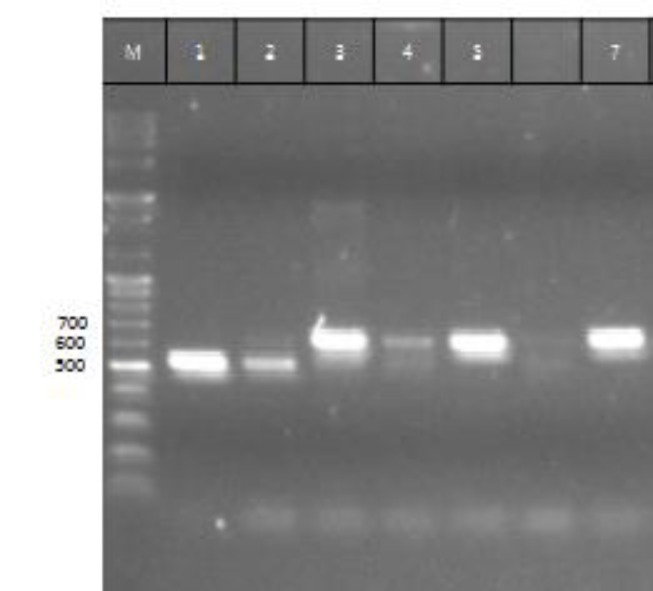
Lane M: DNA ladder (100 bp), lanes 1, 2: *Candida albicans*, lane 3: *C. krusei*, lane 4: *C. glabrata*, lane 5: *C. kefir,* and lane 7: *C. tropicalis*

In the current study, a total of 1,450 urine samples were taken from hospitalized patients (65%) and outpatients (32%) who were suspected of UTI. Initial screening by CHROMagar *Candida* and other conventional methods led to the isolation of *Candida *strains. Out of 500 patients (34.5%) with positive urine culture test, 21 cases (4.2%) were positive for *Candida *strains *Candida albicans* (n=8, 38%), followed by *C. glabrata* (n=6, 28.6%), *C. tropicalis* (n=3, 14.3%), and *C. krusei*/*C.kefir* (n=7, 9.5%), had the highest frequency in both hospitalized and outpatient samples (Table 1). Accordingly, the sequencing results of the strains were consistent with the results of the morphological method. The only exception was *C. dubliniensis*, which was identified in the molecular study as *C.* *albicans* (Figure 1).

In addition, candiduria showed a significant relationship with age and gender. Out of 21 subjects with *Candida* infection, 81% (n=17) of the cases were female (Table 2). The adult women aged 38-53 years constituted the largest group of patients with candiduria. The mean age of the studied subject was 45.7±14.8 years (Table 3). The prevalence rate of UTIs was higher in elderly people. Regarding this, aging, along with gender, can be considered as a predisposing factor for these infections. Some studies have shown that 1 per 5 adult women experience an episode of UTI [15]. In this respect, women who are old or pregnant or have preexisting urinary tract abnormalities or obstruction carry a higher risk of infection. 

**Table 1:** Prevalence rate of *Candida* species

***Candida*** ** species**	**Num.**	**Percentage**	**Colony color**	**Producing germ tubes**	**Beta-glucosidase**
*Candida albicans*	7	33.3	Green or light green	+	+
*Candida glabrata*	6	28.6	Dark pink	-	-
*Candida tropicalis*	3	14.3	Blue purple with a halo around	-	-
*Candida kefir*	2	9.5	Pink round	-	-
*Candida krusei*	2	9.5	Cream	-	-
*Candida dubliniensis*	1	4.7	Dark green	+	-

All patients were examined for the presence of underlying or predisposing factors. Based on the results, they all had at least one predisposing factor contributing to their infection. In this analysis, diabetes (n=8, 21%) was identified as the most frequent predisposing factor for candiduria. Diabetes is accompanied by the appearance of glucose in the urine. The elevation of urine glucose to more than 150 mg/dl sets the ground for the growth of *Candida* strains [19].

The other predisposing factors included the use of the catheter, long-term use of antibiotics, surgery, pregnancy, renal failure, kidney transplant, kidney stones, and use of cytotoxic medications (Table 4). The noteworthy point is that 81% of the patients had more than one predisposing factor for developing the infection. Proteinuria (i.e., protein in urine) and glycosuria (i.e., sugar excretion in urine) were positive in 14 and 12 patients, respectively. However, these two factors cannot be considered alone as risk factors, since they usually appear in the presence of other factors, such as diabetes and kidney stones. 

According to our findings, people with diabetes and patients with predisposing factors are often prone to candiduria. Accordingly, it is useful to consider urine culture for both fungal and bacterial genus by molecular methods in these patients to gain accurate results and adopt a proper treatment. *Candida albicans* is still the most important cause of *Candida *UTIs (Table 1). 

However, non-*albicans Candida* species, such as *C. glabrata, C. krusei, C. parapsilosis,* and *C. tropicalis*, are also important due to their increasing resistance to antifungal agents [22]. Despite the low prevalence of *Candida* UTIs, they have special importance with regard to their potential to induce serious damages to the kidneys and urinary tract system.

**Table 2:** Frequency distribution of the isolated organisms based on gender

**Organism**	**Patient gender**			
	**Male**	**Female**	**Total**	
	**Number**	**Number**	**Number**	**Percentage**
*Candida*	4	15	19	3.8
*Candida* and bacteria	-	2	2	0.4

4.2% of the pataints were positive fot infection with *Candida* species.

**Table 3:** Descriptive indicators of age variable in the studied individuals

**Variable**	**Minimum**	**Maximum**	**Mean**	**Standard Deviation**
Age	18	63	45.7	14.8

The mean age of the studied subjects was 45.7±14.8 years.

**Table 4:** Rate of predisposing factors in patients with candiduria

**Predisposing factors**	**Number of predisposing factors**	**Predisposing factors (%)**
Diabetes	8	21.1
Antibiotic therapy	6	15.7
Pregnancy	2	5.3
Use of catheter	6	15.7
Cytotoxic drug	2	5.3
Kidney stones	2	5.3
Renal failure	3	7.9
Kidney transplant	3	7.9
Surgery	6	15.7

Diabetes is the most important predisposing factor. Other factors in the next rankings include a long-term use of antibiotics, urinary catheter, and surgery.

## Conclusion

In summary, the obtained results demonstrated that despite the increase in the number of UTI cases caused by non *C.albicans* species, this species still ranks first for fungal UTI. In addition, according to the outcomes of the present study, such infections rarely occur in the absence of any predisposing factors. Regarding the results, diabetes (n=8, 21%) was identified as the most frequent risk factor for candiduria.
